# Control of *Rhipicephalus microplus* on taurine cattle with fluralaner in a subtropical region

**DOI:** 10.1186/s13071-024-06200-0

**Published:** 2024-03-01

**Authors:** Tiago Gallina, Camila dos Santos Lagranha, Giancarlo Bilo, Cristiano Malavolta, Lorena Lopes Ferreira, Fernando de Almeida Borges, Daniel de Castro Rodrigues, Tom Strydom, Siddartha Torres, Emmanuel Arnhold, Welber Daniel Zanetti Lopes

**Affiliations:** 1 Federal University of Pampa, Uruguaiana, Rio Grande Do Sul Brazil; 2 Federal University of Minas Gerais, Belo Horizonte, Minas Gerais Brazil; 3 Federal University of Mato Grosso Do Sul, Campo Grande, Mato Grosso Do Sul Brazil; 4 MSD Animal Health, Montes Claros, Brazil; 5 MSD Animal Health, Isando, South Africa; 6 Merck Animal Health, Madison, USA; 7 Federal University of Goiás, Goiânia, Goiás Brazil

**Keywords:** *Bos taurus*, Cattle tick, Isoxazoline, Multiresistant strain, Screwworm

## Abstract

**Background:**

In Rio Grande do Sul, Brazil, a region with a subtropical climate, *Rhipicephalus microplus* is present in taurine cattle raised for beef and milk. In addition, ticks resistant to multiple acaricides are present in this region. Recently, fluralaner (isoxazoline) was launched on the market. Thus, there is a need to evaluate the effects of fluralaner for the control of *R. microplus* on taurine cattle. In addition, occurrence of myiasis by *Cochliomyia hominivorax* larvae after tick parasitism and weight gain of cattle during the experimental period were evaluated.

**Methods:**

Thirty naturally infested cattle were divided into two experimental groups: T01, treated with fluralaner (2.5 mg/kg) pour-on; T02, control. T01 received fluralaner on Days 0 (early summer in January), 42 and 84 (early autumn), whereas T02, a control group, received palliative treatment with a spray formulation when the group mean was ≥ 30 ticks. Counts of *R. microplus* females and calculation of the efficacy of fluralaner were performed on Days 3, 7, 14, 28, 35, 42, 56, 70, 84, 98, 112 and 126. The occurrence of myiasis was assessed throughout the study period. In addition, the weight, weight gain and daily weight gain of the animals were evaluated.

**Results:**

In the 12 evaluations performed, the parasitic load of T01 was near zero. Fluralaner showed 99.5% efficacy on the 3rd day after the first treatment and 100% efficacy from Day 7 to Day 126. *Cochliomyia hominivorax* larvae (*n* = 6; *p* = 0.0251) were found only in the control group (T02). At the end of the study, the animals subjected to treatments with fluralaner gained 32.8 kg more than the animals in the control group.

**Conclusions:**

Application of fluralaner in summer and autumn, with 42-day intervals between treatments, was effective to control *R. microplus* on taurine cattle, which also gained more weight than control cattle. Additionally, no cases of myasis were documented in animals treated with fluralaner.

**Graphical Abstract:**

**Figure:**
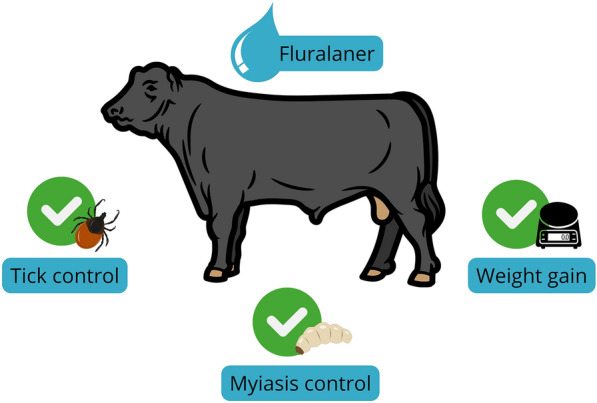

## Background

The tick *Rhipicephalus microplus* causes severe damage to cattle, especially taurine breeds, in tropical and subtropical areas of the world [1]. High parasitic loads increase the risk of transmission of hemoparasites, leather lesions, myiasis and decreased yield due to spoliation [2, 3]. Depending on the temperature and humidity conditions of the region, this tick can complete three to five generations per year [4–7].

In Rio Grande do Sul, southern Brazil, the first generation begins in late winter/early spring with low infestation levels, followed by a second generation in the summer with more ticks and another generation in the autumn (i.e. the third generation) with the highest infestation level [8, 9]. Further studies are needed in this region of Brazil, but some population dynamics studies have demonstrated four peaks of infestation in cattle by this ectoparasite throughout the year [9, 10]. In addition, in this region, both beef and dairy cattle are predominantly taurine [11], which further aggravates the level of cattle infestation by *R. microplus* during the months of the year when the climatic conditions for the development of this ectoparasite are favorable.

The use of acaricides has been the only way to control this tick. This has become another obstacle in the attempt to control this ectoparasite in several countries, including in this region, due to the resistance of *R. microplus* populations to acaricides. The number of chemical treatments required per year to control this tick in southern Brazil is sometimes higher than in other regions [11–14]. This fact has triggered reports of ticks resistant to multiple acaricides, especially in southern Brazil, where resistance to pyrethroids, organophosphates, amidines, phenylpyrazoles, macrocyclic lactones and benzophenylureas has been reported [15–23]. Therefore, before establishing control, it is necessary to consider the epidemiological aspects of this tick in each region and the history of local acaricide resistance [5, 13, 24, 25].

A recent compound belonging to the isoxazoline class, fluralaner, was developed for the control of ectoparasites of dogs, cats and birds as an alternative to other chemical classes on the market, with results showing high efficacy against parasites of these animals in in vivo tests [26–29]. Considering the difficulty of cattle tick control in Rio Grande do Sul, this study aimed to evaluate the control of *R. microplus* in taurine cattle in a region with a subtropical climate using fluralaner to examine the occurrence of myiasis by *Cochliomyia hominivorax* larvae after tick infestation and to evaluate the weight, weight gain and daily weight gain of cattle during the study period.

## Methods

### Study site, animals and experimental groups

The study was conducted from January to May 2021 (summer to autumn, 2nd and 3rd tick generations, respectively, according to Evans [8]) on a commercial farm located in the municipality of Uruguaiana (29°50′09″S, 57°06′35″W), Rio Grande do Sul, southern Brazil. According to the temperature and rainfall criteria for the Köppen climate classification, the city of Uruguaiana can be classified as “Cfa”: (C) humid subtropical zone; (f) oceanic climate, without a dry season and (a) with a hot summer [30].

Thirty male Aberdeen angus cattle that were non-castrated, naturally infested by *R. microplus* and approximately 20 months old, with an average body weight of 315 kg ± 75, were used. These cattle had not received an antiparasitic drug for 70 days prior to the start of the study and were in good health at the beginning of the study. A 20-hectare paddock, also naturally infested by *R. microplus*, was divided into two equal parts with equivalent shady areas, and the cattle were kept separately in the paddocks throughout the experimental period (126 days). During the study, the animals received native pasture as forage and ad libitum mineral supplementation.

The cattle were allocated to the experimental groups on Day 0 and were randomly assigned to the treatments according to a block design. The formation of blocks was performed based on the arithmetic mean of the number of female ticks (measuring 4.5–8.0 mm in length) counted on 3 consecutive days (days -3, -2 and -1), as recommended by Wharton and Utech [31]. The animals were distributed into 15 blocks containing two cattle each and then randomly allocated into one of the groups (T01 and T02) within each block.

### Treatments

Following the premise that tick control should be performed when few ticks are seen on the animals [32, 33], at the beginning of the present study, the animals had an average parasite load of 4.3 ticks/animal. Cattle in group T01 were subjected to three treatments with fluralaner (2.5 mg/kg) pour-on (Exzolt® 5%, MSD Animal Health) on Days 0, 42 and 84 (early summer to early autumn), based on previous results [34]. The interval of 42 days between treatments was determined according to the efficacy results obtained in the registration studies. For animal welfare reasons, animals belonging to T01 would receive palliative treatment if the mean tick counts at any time during the study were ≥ 30 [12]; the palliative treatment consisted of a spray formulation of 125 ppm alphacypermethrin + 400 ppm ethion + 212 ppm chlorpyrifos (Potenty®, MSD, Animal Health). Cattle belonging to T02 were kept as controls. Also for animal welfare reasons, the cattle belonging to this group received this same spray formulation with a combination of pyrethroid and organophosphates whenever the mean tick count of the group was ≥ 30 [12]. All treatments were performed according to the manufacturer’s recommendations.

### Tick counts and efficacy

After dividing the groups, counts of *R. microplus* females measuring between 4.5 and 8.0 mm in length on the left side of each animal and without multiplying by two were performed by the same person and at the same time, according to the methodology proposed by Wharton and Utech [31] on Days 3, 7, 14, 28, 35, 42, 56, 70, 84, 98, 112 and 126.

The acaricidal efficacy was calculated by arithmetic means using the formula recommended by Roulston et al. [35] and adopted by the Brazilian Ministry of Agriculture and Livestock (Ministry of Agriculture, Livestock and Food Supply—MAPA) [36] and Holdsworth et al. [37]:

$${\text{Efficacy}}\,{\text{percentage}} = \left[ {1 - \frac{{{\text{Ta}} \times {\text{Cb}}}}{{{\text{Tb}} \times {\text{Ca}}}}} \right] \times 100$$

where “**Ta**” is the mean number of female ticks (4.5–8 mm) that were counted on treated animals after the treatment; “**Tb**” is the mean number of female ticks (4.5–8 mm) that were counted on treated animals during the 3 days preceding the treatment date; “**Ca**” is the mean number of female ticks (4.5–8 mm) that were counted on untreated control animals after the treatment date; “**Cb**” is the mean number of female ticks (4.5–8 mm) that were counted on untreated control animals during the 3 days preceding the treatment date.

### Detection of myiasis

The occurrence of myiasis caused by *C. hominivorax* larvae was observed in the body of the animals, after infestation by *R. microplus*. The *C. hominivorax* larvae were classified as active (at least one live *C. hominivorax* larva/lesion), which was identified by the number one (1), or nonactive (zero *C. hominivorax* larvae/lesion), which was identified by the number zero (0), as described by Lopes et al. [38].

Animals affected by screwworms received treatment with an oil solution containing 30% dichlorfenthion (Mata Bicheira Coopers®, MSD Animal Health, Brazil) at the wound site. In addition, the larvae were removed from the wound site to confirm the identification of *C. hominivorax* according to the taxonomic criteria described by Spradbery [39].

### Weighing of animals

The animals were weighed individually without previous fasting on D0, D70 and D126. In addition, the animals were also weighed on the dates corresponding to the treatments with fluralaner to adjust the dose of the product used. Weight gain and daily weight gain were calculated for each animal considering differences in body weight during the study (D0, D70 and D126). The scales used for weighing the animals had been tested using a previously known weight.

### Statistical analysis

Data obtained from the counts of *R. microplus* females were log transformed using the equation ln (x + 1) and analyzed in an entirely randomized design within each counting date, as the sphericity and orthogonality tests of the data did not allow a split-plot in-time analysis. The data complied with the assumptions of normality and homogeneity of variances and residuals. Treatment means for tick counts were compared using the F test.

The presence of viable *C. hominivorax* larvae in wounds was analyzed using Fisher's exact test, and differences were considered significant when *P* ≤ 0.05. Regarding body weight, body weight gain and daily body weight gain, the values were compared between the treatments for all evaluation data using the F test. All data were analyzed with a significance level of 5% (*P* ≤ 0.05) using SAS software [40].

## Results

### Tick counts and efficacy

From Day 3 to Day 126 of the study, in the 12 tick counts, the treated group had a lower parasite load (ANOVA, *F*_(1, 28)_ = 2905.57, *P* < 0.001) than the control group. The efficacy of fluralaner was 99.5% on the 3rd day after the beginning of the study and remained at 100% from the 7th to 126th day after the beginning of the study (Table 1), with no need for palliative treatment in animals belonging to this group. On the other hand, it was necessary to perform palliative treatment with the spray solution for the animals in T02 on the 28th, 56th and 98th days of the study, since the cattle in this group had a high parasite load according to the criteria adopted.

**Table 1:** Counts of *Rhipicephalus microplus* females (4.5 to 8 mm in length) present in naturally infested cattle belonging to groups submitted to different control schemes and percentage of effectiveness

Days of the study	Experimental groups/Mean ^1^ number of *R. microplus* females (4.5 to 8 mm)				*P* value	Efficacy (%)
	T01: Treatments with fluralaner		T02: Control (palliative treatment)			T01
0 ^*, α^	4.38	A	4.38	A	0.9861	–
3	0.07	B	13.00	A	< 0.001	99.5
7	0.00	B	8.00	A	< 0.001	100
14	0.00	B	7.73	A	< 0.001	100
28	0.00	B	38.40	A	< 0.001	100
35	0.00	B	12.40	A	< 0.001	100
42 ^α^	0.00	B	2.00	A	< 0.001	100
56 ^β^	0.00	B	62.00	A	< 0.001	100
70	0.00	B	19.00	A	< 0.001	100
84 ^α^	0.00	B	5.67	A	< 0.001	100
98 ^β^	0.00	B	214.20	A	< 0.001	100
112	0.00	B	6.53	A	< 0.001	100
126	0.00	A	14.33	A	< 0.001	100

1: Means followed by the same capital letter in the line do not differ from each other (*p* > 0.05)

^*^Mean counts between − 3, − 2 and − 1 days

^α^Treated group received fluralaner 2.5 mg/kg

^β^Control group received palliative treatment with a spray formulation of 125 ppm alphacypermethrin + 400 ppm ethion + 212 ppm chlorpyrifos

### Myiasis

**Fig. 1:**
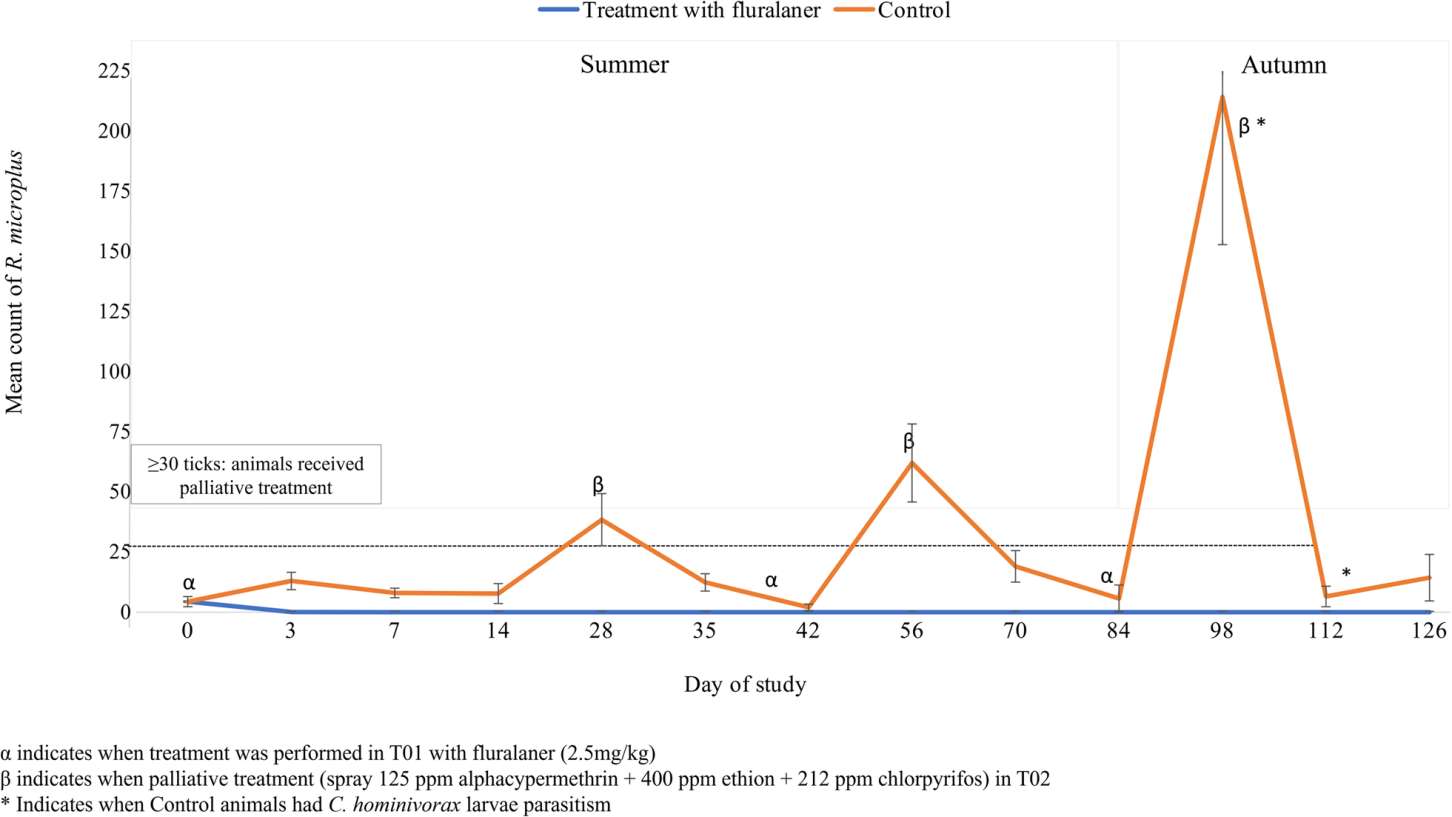
Mean count of *Rhipicephalus microplus* females in naturally infested cattle submitted to different control schemes during 126 study days

Active myiasis was found in 6 of 15 animals of the control group (Fisher's exact test, *P* = 0.01686), which may be attributed to the high parasitic load of *R. microplus* detected in cattle of this group on D98. One animal showed lesions with *C. hominivorax* larvae on D98, and myiasis was observed in the other five animals on D112 of the study (Fig. 1). *Cochliomyia hominivorax* larvae were found in the chest region of infested cattle, where > 30 adult ticks were found. On the same day that the parasites were identified, the larvae were manually removed, and the wounds were treated with 30% dichlorfenthion. No worms were found in animals subjected to control with fluralaner (T01) throughout the study.

### Animal weighing

The results of the average weight, average weight gain and average daily weight gain are described in Table 2. On Days 0, 70 and 126 of the study, the weights of animals in T01 and T02 were homogeneous. There was no difference (ANOVA *F*_(1,28)_ = 3.48, *P* =0.0723) in the mean weight gain from Day 0 to Day 70 of the study between Groups T01 and T02. Between D70 and D126, both groups lost weight. The group treated with fluralaner lost less weight (ANOVA *F*_(1,28)_ = 18.99, *P* < 0.001) than the control group, at -17.5 kg and -39.1 kg, respectively. Over 126 days of study, the animals subjected to control with fluralaner (T01) and the control group (T02) showed average weight gains of 45.1 and 12.3 kg (ANOVA *F*_(1,28)_ = 14.23, *P* < 0.001), respectively. The daily weight gain did not differ from D0 to D70 (ANOVA *F*_(1,28)_ = 3.48, *P* =0.0723) between the fluralaner-treated and control groups. There was daily weight loss between D70 and D126, with a significant difference (ANOVA *F*_(1,28)_ = 18.99, *P* < 0.001) between groups. From D0 to D126, the daily weight gain of the group treated with fluralaner was higher (ANOVA *F*_(1,28)_ = 14.23, *P* < 0.001) than that of the control group, at 0.36 kg and 0.10 kg, respectively.

**Table 2:** Statistical analysis regarding weight, weight gain and daily weight gain of cattle submitted to different control schemes against *Rhipicephalus microplus* during the experimental period of 126 days

Days of study	Variable analyzed	Experimental groups/average weight		*p*-value
		T01: treatments with fluralaner	T02: control (palliative Treatment)	
0	Weight (kg)	307.3	325.9	0.3860
70		369.9	377.3	0.8937
126		352.4	338.2	0.6839
0 to 70	Mean weight gain (kg)	62.5	51.4	0.0723
70 to 126		− 17.5	− 39.1	< 0.001
0 to 126		45.1	12.3	< 0.001
0 to 70	Daily weight gain (kg)	0.89	0.73	0.0723
70 to 126		− 0.25	− 0.55	< 0.001
0 to 126		0.36	0.10	< 0.001

## Discussion

This is the first study conducted with fluralaner in a region of Brazil where there are reports of *R. microplus* resistant to six chemical classes. Fluralaner was highly efficacious against this tick in the evaluated period, with nearly 100% efficacy in all evaluations performed. In addition, animals treated with fluralaner did not exhibit *C. hominivorax* myiasis during the study period and gained more weight than those in the control group.

Although three annual tick generations have been reported in the region where the study was carried out, the challenge created by this ectoparasite in animals may be greater in this location because of the favorable climatic conditions for the development of cattle ticks, especially from the summer (January) through the autumn (April and May) [8, 9]. The trend indicates an increasing risk of cattle infestation by this tick, with the highest load recorded in the autumn, which may have clinical consequences, including mortality due to tick-borne pathogens [41]. For example, in regions where *R. microplus* completes four to five annual generations, four to seven treatments are performed per year [7, 12–14]. In the southern region of Brazil, where the present study was conducted, there are reports of 10 to 12 treatments per year to control *R. microplus* [10]. The reason for this high number of treatments in this region of Brazil may be related to the predominance of taurine cattle, which are highly sensitive to parasitism by *R. microplus* [11], but also to the adoption of an incorrect strategy by farmers.

The data obtained from the cattle in the control group reinforce previous conclusions about the challenge of controlling *R. microplus* ticks on cattle. The animals in the control group were affected by screwworm, especially at the time of year when the tick population increases. On the other hand, no cases of screwworm were observed among cattle treated with fluralaner, even during autumn (May), the period with the highest risk from *R. microplus* in cattle in this region [9, 10]. Similar data were obtained by Reck et al. [3], who found a risk ratio approximately four times greater for an animal to acquire myiasis when highly parasitized by *R. microplus*.

Another parameter evaluated that highlights the importance of the benefits of control with fluralaner was weight gain throughout the study. Notably, there was a marked weight loss between D70 and D126. A possible reason for this finding was that in the second half of the study period, the region was affected by a drought, with days with high temperatures close to 40 °C. This fact could explain this weight loss, since the absence of rain and increase in temperature led to lower forage availability for the herd and fewer hours of grazing, because the animals sometimes sought out shady areas. However, analyzing the whole period, it was possible to verify that the animals treated with fluralaner gained 32.8 kg more than the cattle in the control group. It is well known that tick infestation has a negative effect on the weight gain of parasitized cattle. Studies have shown that each engorged *R. microplus* female is responsible for the loss of 1.37 ± 0.25 g bodyweight in taurine cattle [42]. The presence of gastrointestinal nematodes of cattle that also trigger a decrease in weight gain of animals should be highlighted, according to Zapa et al. [43]. In the present study, the presence of these endoparasites in cattle was not evaluated, and for this reason, the difference in weight gain of the animals should be interpreted with caution and not attributed only to cattle ticks.

Tick control protocols in which ticks are exposed to different chemical classes in each generation, called rotation grazing, is recommended in regions where *R. microplus* has few annual generations, such as where this study was performed. In Rio Grande do Sul, Uruguay and Argentina, a country that borders this region of Brazil, this treatment protocol has already been used and successfully described as a control method [10, 24, 44, 45]. Although the rotational grazing strategy is well studied and established in regions with subtropical climates, this study aimed to evaluate the effect of treatments exclusively with fluralaner to understand how this new molecule behaves in a region with high challenge by *R. microplus* throughout the year. This study does not intend to recommend sequential treatment with fluralaner throughout the year, which would have potential implications in terms of emergence of acaricidal resistance. Nevertheless, our data indicate that fluralaner may be another alternative to include in rotation with other acaricide groups to control *R. microplus* on cattle.

In summary, this study demonstrates important results that should be disseminated among veterinarians and technicians in subtropical climate regions. The regimen of three treatments performed with fluralaner pour-on starting during summer was efficacious to control tick infestation on taurine cattle until the autumn, the time of year in which the levels of *R. microplus* tend to peak in this region. In addition, treatment with the new molecule resulted in the absence of larval infestation due to tick control and greater average weight gain by cattle at the end of the experiment.

## Conclusions

The use of fluralaner pour-on at 42-day intervals was efficacious to control of *R. microplus* on taurine cattle in southern Brazil. In addition, fluralaner prevented the occurrence of screwworms on treated cattle, which also gained more weight compared with untreated cattle.

## Data Availability

The data supporting the findings of this study are included in the manuscript and associated files. Raw data are available from the corresponding author upon request.

## References

[1] Tabor AE, Ali A, Rehman G, Rocha Garcia G, Zangirolamo AF, Malardo T (2017). Cattle Tick *Rhipicephalus microplus*-host interface: a review of resistant and susceptible host responses. Front Cell Infect Microbiol.

[2] Grisi L, Leite R, Martins J, Medeiros A, Andreotti R, Villela HS (2014). Reassessment of the potential economic impact of cattle parasites in Brazil.

[3] Reck J, Marks FS, Rodrigues RO, Souza UA, Webster A, Leite RC (2014). Does *Rhipicephalus microplus* tick infestation increase the risk for myiasis caused by *Cochliomyia hominivorax* in cattle?. Prev Vet Med.

[4] Guglielmone AA, Mangold AJ, Aguirre DH, Gaido AB (1990). Ecological aspects of four species of ticks found on cattle in Salta. Northwest Argentina Vet Parasitol.

[5] Nava S, Mastropaolo M, Guglielmone AA, Mangold AJ (2013). Effect of deforestation and introduction of exotic grasses as livestock forage on the population dynamics of the cattle tick *Rhipicephalus* (*Boophilus*) *microplus* (Acari: Ixodidae) in northern Argentina. Res Vet Sci.

[6] Cruz BC, de Lima Mendes AF, Maciel WG, dos Santos IB, Gomes LVC, Felippelli G (2020). Biological parameters for *Rhipicephalus microplus* in the field and laboratory and estimation of its annual number of generations in a tropical region. Parasitol Res.

[7] Nicaretta JE, Zapa DMB, Couto LFM, Heller LM, Cavalcante ASA, Cruvinel LB (2021). *Rhipicephalus microplus* seasonal dynamic in a Cerrado biome, Brazil: An update data considering the global warming. Vet Parasitol.

[8] Evans DE (1992). Tick infestation of livestock and tick control methods in Brazil: A situation report. Int J Trop Insect Sci.

[9] Martins JR, Evans DE, Cereser VH (2002). Partial strategic tick control within a herd of European breed cattle in the state of Rio Grande do Sul, southern Brazil. Exp Appl Acarol.

[10] Centenaro FC, Barbieri A, Rico IB, Gonchoroski GZ, Jardim FT, Doyle RL (2022). Rotational and selective protocols using acaricides to control a multi-resistant strain of *Rhipicephalus microplus* under field conditions in Southern Brazil. Ticks Tick Borne Dis.

[11] Zarth PA. Introdução de novas raças de gado no sul do Brasil (1870–1950). Revista História: Debates e Tendências. 2016;16.

[12] Costa Gomes LV, Lopes WDZ, Teixeira WFP, Maciel WG, Cruz BC, Felippelli G (2016). Population dynamics and evaluation of the partial selective treatment of crossbreed steers naturally infested with *Rhipicephalus* (*Boophilus*) *microplus* in a herd from the state of Minas Gerais in Brazil. Vet Parasitol.

[13] Felippelli G, Teixeira WFP, Gomes LVC, Maciel WG, Cruz BC, Buzzulini C (2022). Tick infestation level interferes with spray formulation (organophosphate + pyrethroid) efficacy against *Rhipicephalus microplus*. Ticks Tick Borne Dis.

[14] Gomes LVC, Teixeira WFP, Maciel WG, Felippelli G, Buzzulini C, Soares VE (2022). Strategic control of cattle co-parasitized by tick, fly and gastrointestinal nematodes: Is it better to use ecto + endoparasiticide or just endectocide formulations?. Vet Parasitol.

[15] Fernández-Salas A, Rodríguez-Vivas RI, Alonso-Díaz MÁ (2012). Resistance of *Rhipicephalus microplus* to Amitraz and Cypermethrin in Tropical Cattle Farms in Veracruz. Mexico Journal of Parasitology.

[16] Cuore U, Solari MA (2014). Multiresistant Population of *Rhipicephalus* (Boophilus) *Microplus* Ticks in Uruguay. Veterinaria.

[17] Reck J, Klafke GM, Webster A, DallAgnol B, Scheffer R, Souza UA (2014). First report of fluazuron resistance in *Rhipicephalus microplus*: A field tick population resistant to six classes of acaricides. Vet Parasitol.

[18] Souza Higa LO (2015). Acaricide Resistance Status of the *Rhipicephalus microplus* in Brazil: a literature overview. Med Chem.

[19] Klafke G, Webster A, Dall Agnol B, Pradel E, Silva J, de La Canal LH (2017). Multiple resistance to acaricides in field populations of *Rhipicephalus microplus* from Rio Grande do Sul state. Southern Brazil Ticks Tick Borne Dis.

[20] Fular A, Sharma AK, Kumar S, Nagar G, Chigure G, Ray DD (2018). Establishment of a multi-acaricide resistant reference tick strain (IVRI-V) of *Rhipicephalus microplus*. Ticks Tick Borne Dis.

[21] Rodriguez-Vivas RI, Jonsson NN, Bhushan C (2018). Strategies for the control of *Rhipicephalus microplus* ticks in a world of conventional acaricide and macrocyclic lactone resistance. Parasitol Res.

[22] Vilela VLR, Feitosa TF, Bezerra RA, Klafke GM, Riet-Correa F (2020). Multiple acaricide-resistant *Rhipicephalus microplus* in the semi-arid region of Paraíba State. Brazil Ticks Tick Borne Dis.

[23] Dzemo WD, Thekisoe O, Vudriko P (2022). Development of acaricide resistance in tick populations of cattle: A systematic review and meta-analysis. Heliyon.

[24] Nava S, Toffaletti JR, Morel N, Guglielmone AA, Mangold AJ (2019). Efficacy of winter–spring strategic control against *Rhipicephalus* (*Boophilus*) *microplus* infestations on cattle in an area with ecological conditions highly favourable for the tick in northeast Argentina. Med Vet Entomol.

[25] Nicaretta JE, Couto LFM, Heller LM, Ferreira LL, Cavalcante ASA, Zapa DMB (2021). Evaluation of different strategic control protocols for *Rhipicephalus microplus* on cattle according to tick burden. Ticks Tick Borne Dis..

[26] Walther FM, Allan MJ, Roepke RK, Nuernberger MC (2014). Safety of fluralaner chewable tablets (Bravecto™), a novel systemic antiparasitic drug, in dogs after oral administration. Parasit Vectors.

[27] Huyghe B, Le Traon G, Flochlay-Sigognault A (2017). Safety of fluralaner oral solution, a novel systemic poultry red mite treatment, for chicken breeders’ reproductive performances. Parasit Vectors.

[28] Ranjan S, Young D, Sun F (2018). A single topical fluralaner application to cats and to dogs controls fleas for 12 weeks in a simulated home environment. Parasit Vectors.

[29] Chiummo R, Petersen I, Plehn C, Zschiesche E, Roepke R, Thomas E (2020). Efficacy of orally and topically administered fluralaner (Bravecto®) for treatment of client-owned dogs with sarcoptic mange under field conditions. Parasit Vectors.

[30] Alvares CA, Stape JL, Sentelhas PC, de Moraes Gonçalves JL, Sparovek G (2013). Köppen’s climate classification map for Brazil. Meteorol Z.

[31] Wharton RH, Utech KBW (1970). The relation between engorgement band dropping of *Boophilus microplus* (Canestrini) (Ixodidae) to the assessment of tick numbers on cattle. Aust J Entomol.

[32] Furlong J (1993). Controle do carrapato dos bovinos na Região Sudeste do Brasil. Cadernos Técnicos da Escola de Veterinária da UFMG.

[33] Andreotti R, García MV, Koller WW. Carrapatos na cadeia produtiva de bovinos. Empresa Brasileira de Pesquisa Agropecuária. Ministério da Agricultura Pecuária e Abastecimento. Brasília; 2019.

[34] Da Costa AJ, de Souza Martins JR, de Almeida Borges F, Vettorato LF, Barufi FB, de Oliveira Arriero Amaral H (2023). First report of the efficacy of a fluralaner-based pour-on product (Exzolt^®^ 5%) against ectoparasites infesting cattle in Brazil. Parasit Vectors.

[35] Roulston WJ, Wharton RH (1967). Acaricide tests on the biarra strain of organophosphorus resistant cattle tick *Boophilus microplus* from southern queensland. Aust Vet J.

[36] Brazil, 1997. Ministério da Agricultura e do Abastecimento. Secretaria de Defesa Agropecuária, Portaria n. 48, 12/05/1997.

[37] Holdsworth P, Rehbein S, Jonsson NN, Peter R, Vercruysse J, Fourie J (2022). World Association for the Advancement of Veterinary Parasitology (WAAVP) second edition: Guideline for evaluating the efficacy of parasiticides against ectoparasites of ruminants. Vet Parasitol.

[38] Lopes WDZ, Teixeira WFP, Felippelli G, Cruz BC, Maciel WG, de Matos LVS (2013). Ivermectina e abamectina em diferentes doses e vias de aplicação contra larvas de *Cochliomyia hominivorax* em bolsas escrotais de bovinos recém-castrados, provenientes da região sudeste do Brasil. Ciência Rural.

[39] Spradbery JP. A manual for the diagnosis of screw-worm fly. Department of Agriculture F& F, editor. Canberra, Australia; 2002.

[40] SAS User´s Guide. Statistics. SAS Institute Inc. 2016.

[41] de Almeida MB, Tortelli FP, Riet-Correa B, Ferreira JLM, Soares MP, Farias NAR (2006). Tick fever in southern Brazil: a retrospective study of 1978–2005. Pesq Vet Bras.

[42] Jonsson NN (2006). The productivity effects of cattle tick (*Boophilus microplus*) infestation on cattle, with particular reference to *Bos indicus* cattle and their crosses. Vet Parasitol.

[43] Zapa DMB, Couto LFM, Heller LM, Cavalcante ASA, Nicaretta JE, Cruvinel LB (2021). Association between fecal egg count and weight gain in young beef cattle. Livest Sci.

[44] Nava S, Mangold AJ, Canevari J, Morel N, Guglielmone AA (2014). Strategic treatments with systemic biocides to control *Rhipicephalus* (Boophilus) *microplus* in northwestern Argentina. InVet.

[45] Morel N, Signorini ML, Mangold AJ, Guglielmone AA, Nava S (2017). Strategic control of *Rhipicephalus* (*Boophilus*) *microplus* infestation on beef cattle grazed in *Panicum maximum* grasses in a subtropical semi-arid region of Argentina. Prev Vet Med.

